# Identification of *Gyromitra infula*: A Rapid and Visual Method Based on Loop-Mediated Isothermal Amplification

**DOI:** 10.3389/fmicb.2022.842178

**Published:** 2022-02-18

**Authors:** Xiaomei Xie, Bu Li, Yuguang Fan, Renhe Duan, Chonghua Gao, Yuan Zheng, Enjing Tian

**Affiliations:** ^1^Engineering Research Center of Edible and Medicinal Fungi, Ministry of Education, Jilin Agricultural University, Changchun, China; ^2^Key Laboratory of Tropical Translational Medicine of Ministry of Education, College of Pharmacy, Hainan Medical University, Haikou, China

**Keywords:** mushroom poisoning, *Gyromitra infula*, loop-mediated isothermal amplification, internal transcribed spacer, onsite rapid detection

## Abstract

With mushroom poisoning emerging as one of the most serious food safety problems worldwide, a rapid identification method of poisonous mushrooms is urgently required to investigate the source of poisoning. *Gyromitra infula*, a kind of poisonous mushroom, contains gyromitrin toxin, which causes epileptogenic neurotoxicity and hemolytic disease. This study aimed to establish a rapid and visual method of *G. infula* identification based on loop-mediated isothermal amplification (LAMP). A set of specific LAMP primers was designed, and its specificity in *G. infula* was confirmed against various mushroom species, including its closely related species and other macrofungi. The sensitivity assay showed that the minimum concentration of genomic DNA detected by LAMP was 1 ng/μl. The method’s applicability was conducted by preparing mushroom samples that were boiled and digested in artificial gastric juice. The results showed that the content as low as 1% *G. infula* can be successfully detected. This method can be completed within 90 min, and the reaction results can be directly observed by the naked eyes. Hence, the identification method of *G. infula* established based on LAMP in this study is accurate, rapid, sensitive, and low-cost, which is required for clinical treatment or forensic analysis when mushroom poisoning occurs.

## Introduction

Mushrooms are widely distributed globally, some of which have high nutritional value and medicinal efficacy (Lei et al., 2020), but some of which are poisonous. There are more than 4,000 mushrooms species in China, among which 966–1,020 are edible (Dai et al., 2010; Wu et al., 2019), 692 are medicinal (Wu et al., 2019), and 435–480 are poisonous (Bau et al., 2014; Wu et al., 2019). Poisonous mushrooms with toxins can make people and animals have toxic reactions and even lead to death. Mushroom poisoning mostly occurs from June to October annually. Mushroom poisoning is mostly common in Southwest China and Central China, followed by Southern China, Eastern China, Northern China, Northeast China, and Northwest China (Chen, 2014; Chen et al., 2016). Although poisonous mushrooms can cause poisoning, they play a certain role in some fields, such as medicine and insecticides, due to their biologically active compounds (Wu et al., 2019; Sheikha, 2021).

Many poisonous mushrooms have similar morphology as edible mushrooms, and it is difficult to distinguish between them through observation (Yang, 2013). Among these mushrooms, *Gyromitra infula* (Schaeff.) Quél is commonly mistaken as an edible mushroom, and is distributed in different regions of China during summer and autumn. *Gyromitra infula* is extremely similar to edible *Helvella* species (such as *Helvella macropus*) and tend to be confused with the latter, leading to accidental ingestion and poisoning. *Gyromitra infula* contains the toxin gyromitrin, which is converted to monomethylhydrazine in human body, one of the raw materials of rocket fuel (Pan et al., 2012). Additionally, *G. infula* may be carcinogenic if accidentally ingested. If *G. infula* is accidentally ingested, it can lead to epileptogenic neurotoxicity and hemolytic disease (Diaz, 2005; Chen et al., 2016), characterized by early symptoms, such as headache, diarrhea, nausea, and vomiting; 2 days after ingestion, the patient will develop chills, abdominal pain, hepatosplenomegaly, fever, vertigo, delirium, seizures, coma, and hemoglobinuria. In severe cases, patients may die of acute kidney failure. Gyromitrin and the hydrolyzed product monomethylhydrazine in these mushrooms can lyse and destroy erythrocytes, leading to acute hemolysis. Rapid detection and identification of poisonous mushrooms (such as *G. infula*) when mushroom poisoning occurs is essential and urgent for investigating mushroom poisoning and treatment of poisoning patients (Li et al., 2020). Conventional detection methods such as morphology identification, high-performance liquid chromatography, and polymerase chain reaction (PCR; Ahmed et al., 2010; Yao et al., 2018; Wurita et al., 2019; Cevik, 2020) are not suitable for regions that are economically underdeveloped and have a lack of poisonous mushroom knowledge and equipment limitations. Therefore, there is an urgent need for a new, simple, practical, and cheap rapid detection method for *G. infula* that can be used for onsite applications at the grassroots level.

Several test methods have been derived from PCR, such as real-time fluorescence PCR, PCR-restriction fragment length polymorphism (PCR-RFLP), and DNA barcoding (Sugano et al., 2017; Sheikha and Hu, 2018; Sheikha et al., 2018; Kondo et al., 2019; Wu et al., 2019). However, these methods depend on expensive equipment, are time-consuming and cannot be used in rapid onsite detection. Loop-mediated isothermal amplification (LAMP) is a new nucleic acid amplification method developed by Notomi et al. (2000). In LAMP, two pairs of specific primers (one pair of outer primers and one pair of inner primers) are designed for six regions in the target gene, and a strand-displacement DNA polymerase (Bst DNA polymerase) can be used for amplification at a constant temperature (around 65°C), where nucleic acids can be amplified to a magnitude of 10^9^–10^10^ within 1 h. The third primer pair (loop primer) can be selectively added to the system to accelerate the reaction and shorten reaction time (Yu et al., 2011). Compared to routine PCR, LAMP has a higher specificity and sensitivity (10 times compared to that of PCR) and does not require template denaturation, long periods of thermal cycling, cumbersome electrophoresis, and ultraviolet observation. Result detection is also extremely simple, where the amplification will simultaneously produce red and yellow changes, of which, a yellow indicates a positive result, and a pink indicates a negative result, which can be observed with only the naked eye, does not require a PCR cycler and other expensive equipment, and only a thermostatic water bath is necessary to conduct the reaction (Xiang et al., 2019). With all abovementioned advantages, LAMP has a high specificity and sensitivity, is easy to operate, does not require expensive reagents and equipment, and is extremely suitable for rapid detection and onsite application at the grassroots level (Kuang et al., 2007). As the technology of LAMP gradually matures, it is widely used in fields such as food safety testing (Zhu and Wang, 2018), testing of pathogenic microorganisms (Lv et al., 2020), environmental testing (Becherer et al., 2020), and clinical disease diagnosis (Gu et al., 2021). In the identification of mushroom species, Vaagt et al. (2013) were the first to employ LAMP to detect *Amanita phalloides*. He et al. (2019) used LAMP to detect 10 highly toxic species of *Amanita*. Besides the Amanita genus, Wang et al. (2021) used LAMP to detect *Chlorophyllum molybdites*. However, there is a lack of effective and rapid identification methods for *G. infula*.

In this study, an accurate, visual, rapid, sensitive and low-cost method was established for the detection of *G. infula* based on LAMP, which can be used in resource-poor areas without expensive and complex instruments. This method is of great significance for the rapid identification of poisonous mushrooms in poisoning incidents, the targeted treatment after poisoning and the prevention of mushroom poisoning.

## Materials and Methods

### Experimental Materials

**Figure 1:**
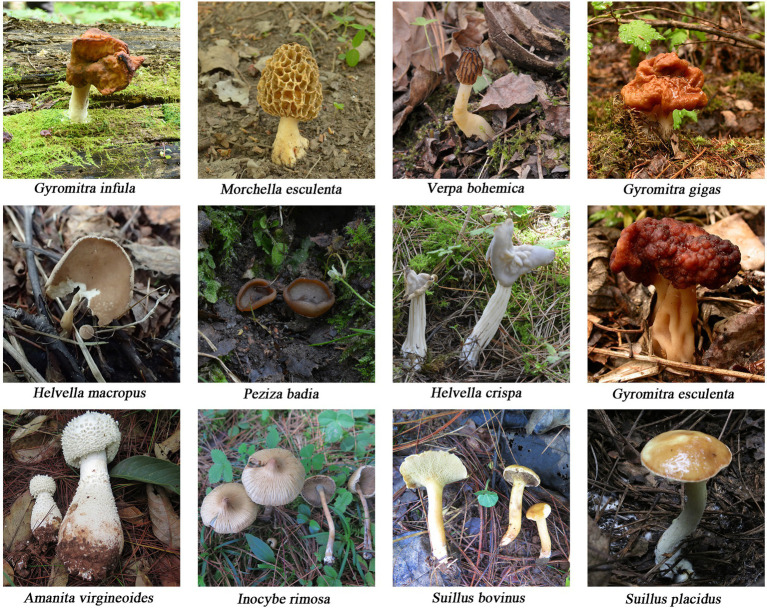
Photographs of mushroom species collected in this study. Photos by Yuguang Fan, Enjing Tian, and Qianqi Ye.

The mushroom materials used in this experiment were collected from the field, and the specimens were deposited in Herbarium Mycology of Jilin Agriculture University (HMJAU). Table 1 shows the sample information, and Figure 1 shows the fruiting bodies. All samples were identified accurately based on morphology (Singer, 1975; Tian and Matheny, 2021), and the identification results were validated through the molecular biology analysis.

**Table 1:** Information of mushrooms used in *Gyromitra infula* LAMP experiments.

Specimen number	Species name	Locality	Toxic or not	GenBank number (ITS)
HMJAU 37501/G90316	*Gyromitra infula*	Heilongjiang, China	Yes	OL678121
HMJAU 37502/072308	*Helvella crispa*	Guizhou, China	Yes	OL678122
HMJAU 22166	*Gyromitra gigas*	Jilin, China	Yes	OL678123
HMJAU 37503/072302	*Suillus placidus*	Guizhou, China	Yes	OL678124
HMJAU 37504/072338	*Inocybe rimosa*	Guizhou, China	Yes	OL678125
HMJAU 37539	*Amanita virgineoides*	Yunnan, China	Yes	OL678127
HMJAU 37505/071502	*Suillus bovinus*	Yunnan, China	Yes	OL678126
HMJAU 9531	*Verpa bohemica*	Jilin, China	Yes	OL678128
HMJAU 21658	*Peziza badia*	Heilongjiang, China	Yes	OL678129
HMJAU 37508/081546	*Morchella esculenta*	Jilin, China	No	OL678132
HMJAU 37507/FYG20160510	*Gyromitra esculenta*	Hainan, China	Yes	OL678131
HMJAU 37506/Y090607	*Helvella macropus*	Jilin, China	No	OL678130

### DNA Extraction, PCR Amplification, and Sequencing

About 30 mg of every sample were placed in a 1.5 ml centrifuge tube with 2–3 steel beads added. The samples were dried in liquid nitrogen and homogenized into powder. Following that, the DNA secures new plant genomic DNA extraction kit (TIAGEN, China) was used for total genomic DNA extraction. NanoDrop 2000 ultra-spectrophotometer was used to measure DNA concentration and purity. After measurement, DNA concentration was diluted to 10 ng/μl for further study. The ITS1F/ITS4 universal primer (Gardes and Bruns, 1993) was used for PCR amplification of 12 mushrooms in a PCR thermal cycler. The PCR mixtures consisted of 12.5 μl 2 × Taq Master Mix, 1 μl of each of the two primers, 2 μl DNA template, and 8.5 μl ddH_2_O for a total volume of 25 μl. The ITS amplification condition was initial denaturation at 94°C for 4 min, followed by 30–34 cycles of 94°C 40 s, 55°C 40 s, 72°C 50 s, and then a final extension at 72°C for 10 min. The 2% agarose gel electrophoresis was used to examine the amplification products. After that, the products were directly sent to Sangon Biotech Co. Ltd. (Shanghai) for sequencing. The Sequencher 5.4.5 (Gene Codes, Ann Arbor, Michigan) was used to assemble the two-way sequencing results, remove the poor fragments of the sequences, check the accuracy of every base, and manually correct the sequencing results according to sequence chromatograms. And then, these sequences were uploaded to National Center for Biotechnology Information (NCBI) for BLAST analysis to validate the accuracy of morphological identification based on sequence similarity and comprehensive score.^1^ Finally, DNA sequences generated in this study were deposited in GenBank, and the accession numbers are listed in Table 1.

### LAMP Primer Design and Screening

**Figure 2:**
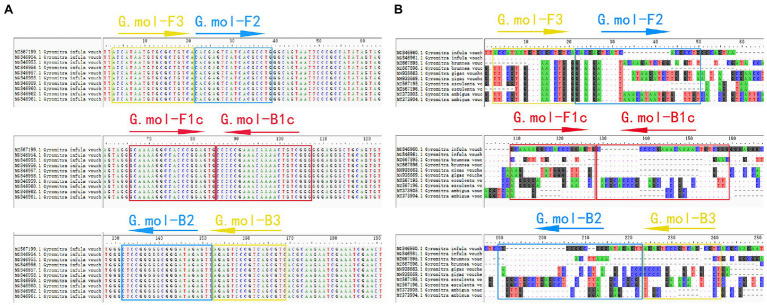
The LAMP primer set of *Gyromitra infula* designed in the ITS region. **(A)** The multiple-sequence alignment of *Gyromitra infula* homologous species. **(B)** The multiple-sequence alignment of *Gyromitra infula* similar or related species (not all shown in the picture).

The ITS gene sequence was used as amplification targets to design *G. infula* LAMP-specific primer pairs. Ten *G. infula* ITS sequences (accession numbers: MZ567199, MG846961, MG846962, MG846960, MG846959, MG846958, MG846957, MG846956, MG846955, and MG846954) were downloaded from NCBI GenBank, and the multi-sequence alignment was conducted (Figure 2A) to search for conserved regions in *G. infula* for primer design. To ensure primer specificity, the ITS sequences of four other *Gyromitra* species phylogenetically close to *G. infula* (*G. gigas*, *G. esculenta*, *G. ambigua*, and *G. brunnea*) and the mushroom sample sequences obtained in this study were used for multi-sequence alignment using the AliView 1.17 (Larsson, 2014) software (Figure 2B). Inter-species differentiated and intra-species conserved regions were used as primer binding sites for the LAMP primer design. GLAPD (Jia et al., 2019) was used for the online design of specific primers.^2^ Ring primers were added in order to further shorten the reaction time (Nagamine et al., 2002). Firstly, the preliminary LAMP primer sets were obtained through the system software calculation. Then, a theoretical verification was carried out by Primer-BLAST on NCBI for specificity of LAMP primers.^3^ We assessed the primers characteristics, including the primer dimers, hairpins, false priming using the Primer Premier 5.0 software. Following that, two sets of primers (primer set I and primer set II, Table 2) were obtained. Finally, primer set I was essentially selected as the most suitable primer set using experimental screening, which included two outer primers (G.mol-F3 and G.mol-B3), four inner primers (G.mol-F1c, G.mol-F2, G.mol-B1c, and G.mol-B2), and one loop primer (G.mol-LB).

**Table 2:** Primer sets for *Gyromitra infula* LAMP testing.

Primer sets	Primers	Sequence(5'-3')
I	G.mol-F3	ACCATAATGTGCGCTGTCA
	G.mol-F2	CACGAGTCATCACGCCTG
	G.mol-F1c	CACTCCGGGTGGCCTTTTGC
	G.mol-B3	GACGCTGACGGGACTCT
	G.mol-B2	AACTCTATCCCGCCCCCG
	G.mol-B1c	CCCCCGAAACAAAACTGTCGGG
	G.mol-LB	GGGAGGGCTGCAGTGTCAG
II	G.mol-F3’	GTCCACCTGAAACACAACA
	G.mol-F2’	CACCAAACTGCAGTCAGA
	G.mol-F1c’	CCAAGAGATCCGTTGTTGAAAGTT
	G.mol-B3’	TTCCCGCATCGATGAAGAACG
	G.mol-B2’	ATGATTCACTGAATTCTGCAA
	G.mol-B1c’	TGTGCGTTCAAAGATTCG
	G.mol-LB’	GCGAAATGCGATAAGTAATGTGAA

### LAMP Reaction and Specificity Test of the Primer Set

There were 10 μl in the LAMP reaction system, which included 5 μl LAMP master mix (2×; purchased from New England Biolabs, United States), 3 μl primer mixture (the concentrations of forward outer primer F3 and reverse outer primer B3 were 0.6 μmol/L, respectively; the concentrations of the forward inner primer F2, reverse inner primer B2, forward inner primer F1c, and reverse inner primer B1c were 4.8 μmol/L, respectively, and the concentration of the loop primer LB was 2 μmol/L), 2 μl DNA template, and topped up with ddH_2_O to 10 μl. The LAMP reaction was conducted at a constant temperature of 65°C in a PCR thermal cycler, and the reaction time was 60–90 min. The reaction tube was placed on a piece of white paper after the reaction, and the reaction result was determined based on the color change. The result is determined to be positive when the color of the tube changes from pink to yellow by visual observation under visible light. To test the specificity of LAMP primer set, a positive control (*G. infula*), 11 negative controls (including species that are closely related to *G. infula*, easily confused species with similar morphology, and other common poisonous and edible mushrooms. See Table 1 for the species information), and one blank control (ddH_2_O) were used for the LAMP reaction. If the color of the *G. infula* (positive control) tube changes from pink to yellow, while the negative control and blank control tubes remained pink, this indicates that the primer set has a good specificity.

### Applicability Test of LAMP Method

To further validate the applicability of LAMP primer set I in Table 2, we simulated the mushroom processing and human digestion processes. Boiled and digested *G. infula* and *G. infula* mixture were used for testing.

Firstly, *G. infula* and *Morchella esculenta* were mixed in a mass ratio of 1:1, 1:9, and 1:99 to prepare the *G. infula* mixture. Following that, the *G. infula* and *M. esculenta* mixture was boiled at 100°C for 10 min. Finally, DNA extraction and LAMP reaction were conducted, and changes in the color of the solution were observed after the reaction.

The artificial gastric solution was composed of 0.05 g potassium chloride, 0.42 g sodium chloride, and 0.32 g pepsin and topped up with 100 ml water. Following that, 1 mol/l hydrochloric acid was used to adjust the pH to 3.0 (Minekus et al., 2014). The boiled *G. infula* and *G. infula* mixture (*G. infula* and *M. esculenta* mixed in a mass ratio of 1:1, 1:9, and 1:99) were incubated in an artificial gastric solution at 37°C for 4 h. Then, DNA extraction and LAMP reaction were conducted and changes in the color of the solution were observed after the reaction.

## Results

### Specificity Analysis

**Figure 3:**
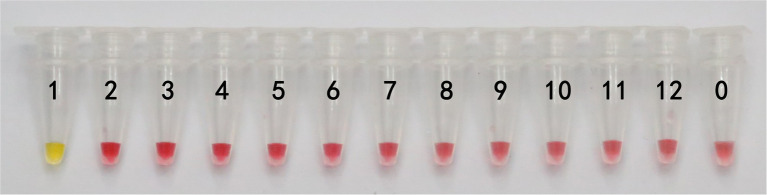
The specificity results of the LAMP primer set I of *Gyromitra infula.* The specificity of primer set I was tested with *Gyromitra infula* and 11 other related species. 1. *Gyromitra infula*; 2. *Helvella crispa*; 3. *Gyromitra gigas*; 4. *Suillus placidus*; 5. *Inocybe rimosa*; 6. *Amanita virgineoides*; 7. *Suillus bovinus*; 8. *Gyromitra esculenta*; 9. *Verpa bohemica*; 10. *Peziza badia*; 11. *Morchella esculenta*; 12. *Helvella macropus*; 0. ddH_2_O.

**Figure 4:**
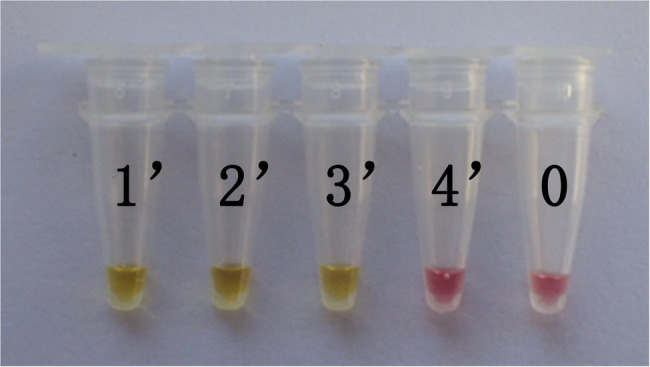
The test results of the LAMP primers set II. The specificity of primer set I was further tested through the control primer set II. The LAMP samples included *Gyromitra infula* and 3 other non-target species. 1'. *Gyromitra infula*; 2'. *Helvella crispa*; 3'. *Gyromitra gigas*; 4'. *Suillus placidus*; 0. ddH_2_O.

In this study, DNA from *G. infula* and 11 other species (including closely related species, morphologically similar species, and other poisonous and edible mushrooms) were used as templates. Table 1 shows the species information. Primer set I in Table 2 was used for the LAMP experiment. Figure 3 and Table 3 show the specificity validation results, where only the reaction tube no. 1 (*G. infula*) showed significant color change (from pink to yellow), proving that successful amplification occurred with the designed primers. The other 11 negative control and blank control tubes remained pink, showing no cross reaction occurred. In this experiment, we carried out three replicates and each replicate showed the same result. This further proved that the LAMP primer set in this experiment only amplified the DNA of *G. infula*. There was no nonspecific amplification of closely related species, morphologically similar species, and other poisonous and edible mushrooms. To further validate the specificity of primer set I, the control primer set II in Table 2 was used for the LAMP control experiment of *G. infula* and closely related species. Three replicates were conducted in the control experiment. As shown in Figure 4 and Table 4, besides the blank control and No. 4' (*Suillus placidus*) tube that did not show a color change, other samples (1' *G. infula*, 2' *Helvella crispa*, and 3' *G. gigas*) were positive. This showed that the primer set II in Table 2 can amplify other non-target species in addition to *G. infula* and was not suitable for LAMP testing for *G. infula*.

**Table 3:** Specificity results of the LAMP primer set I of *Gyromitra infula.*

Number	Species name	Locality	LAMP results
1	*Gyromitra infula*	Heilongjiang, China	+
2	*Helvella crispa*	Guizhou, China	−
3	*Gyromitra gigas*	Jilin, China	−
4	*Suillus placidus*	Guizhou, China	−
5	*Inocybe rimosa*	Guizhou, China	−
6	*Amanita virgineoides*	Yunnan, China	−
7	*Suillus bovinus*	Yunnan, China	−
8	*Gyromitra esculenta*	Xinjiang, China	−
9	*Verpa bohemica*	Jilin, China	−
10	*Peziza badia*	Heilongjiang,China	−
11	*Morchella esculenta*	Jilin, China	−
12	*Helvella macropus*	Hainan, China	−
0	ddH_2_O		−

“+” is positive and “−” is negative.

**Table 4:** The test results of the LAMP primers set II.

Number	Species name	Locality	LAMP results
1’	*Gyromitra infula*	Heilongjiang, China	+
2’	*Helvella crispa*	Guizhou, China	+
3’	*Gyromitra gigas*	Jilin, China	+
4’	*Suillus placidus*	Guizhou, China	−
0	ddH_2_O		−

“+” is positive and “−” is negative.

### Sensitivity Analysis

**Figure 5:**
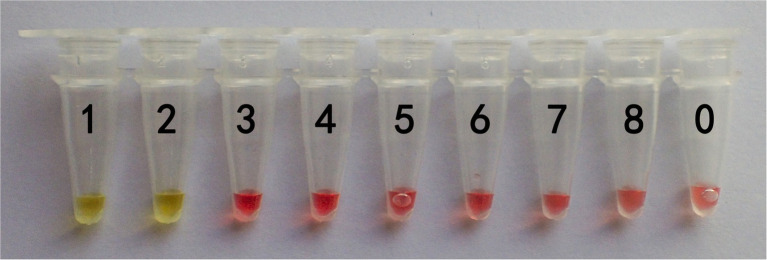
Sensitivity results of the LAMP primers set I of *Gyromitra infula.* The minimum DNA concentration detected was evaluated by using a series of *Gyromitra infula* DNA dilutions. 1: 10 ng/μl; 2: 1 ng/μl; 3: 0.1 ng/μl; 4: 0.01 ng/μl; 5: 1 pg/μl; 6: 0.1 pg/μl; 7: 0.01 pg/μ; 8: 1 fg/μl; 0: ddH_2_O. The color changing to yellow indicated a positive reaction.

The lowest DNA concentration detected is known as sensitivity. Therefore, the genomic DNA concentration of *G. infula* was serially diluted 10-fold from 10 ng/μl to 1 fg/μl (Table 5) for the LAMP reaction, and color changes after the reaction were observed. Three replicates were performed in the sensitivity experiment. As shown in Figure 5 and Table 5, tubes 1 and 2 changed from pink to yellow. The limit of detection of the LAMP reaction was 1 ng, which was far lower than many conventional detection methods and sufficient to meet the actual detection needs with high sensitivity.

**Table 5:** Sensitivity results of the LAMP primers set of *Gyromitra infula.*

Number	*Gyromitra infula* concentration	LAMP results
1	10 ng/μl	+
2	1 ng/μl	+
3	0.1 ng/μl	−
4	0.01 ng/μl	−
5	1 pg/μl	−
6	0.1 pg/μl	−
7	0.01 pg/μl	−
8	1 fg/μl	−
0	ddH_2_O	−

“+” is positive and “−” is negative.

### Applicability Analysis

**Figure 6:**
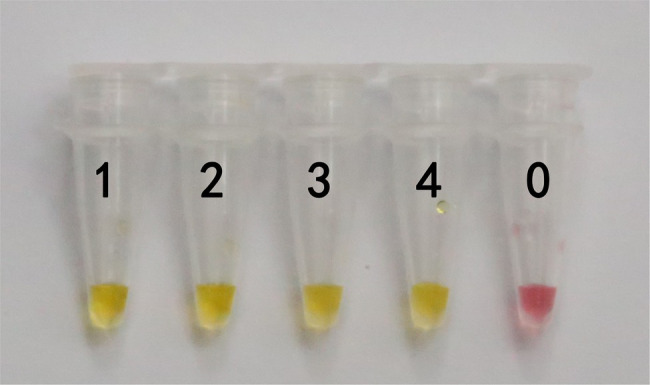
Applicability results of LAMP assay with boiled mushrooms. Applicability analysis of the LAMP method established in this study was evaluated by using boiled mushrooms to simulate the processing of mushrooms. 1. *Gyromitra infula.* 2. 50% *Gyromitra infula* + 50% *Morchella esculenta*; 3. 10% *Gyromitra infula* + 90% *Morchella esculenta*; 4. 1% *Gyromitra infula* + 99% *Morchella esculenta*; 0. ddH_2_O.

**Figure 7:**
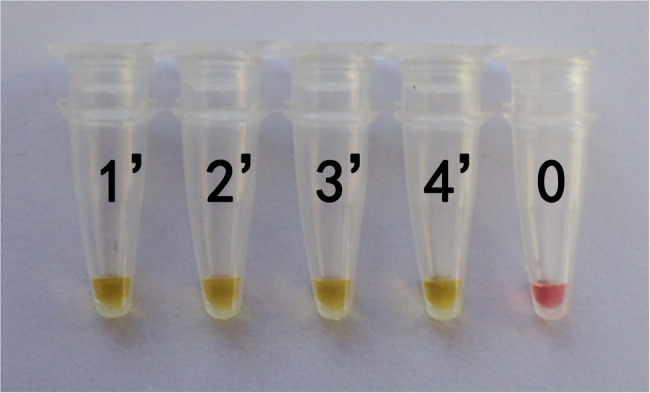
Applicability results of LAMP assay with digested mushrooms after boiled. Applicability analysis of the LAMP method established in this study was evaluated by using digested mushrooms after boiled to simulate the digestion of mushrooms. 1'. *Gyromitra infula.* 2'. 50% *Gyromitra infula* + 50% *Morchella esculenta*; 3'. 10% *Gyromitra infula* + 90% *Morchella esculenta*; 4'. 1% *Gyromitra infula* + 99% *Morchella esculenta*; 0. ddH_2_O.

In actual application, the tested mushroom materials usually undergo processing to a certain degree or are in some abnormal states. For example, humans often cook mushrooms before consumption. When poisonous mushrooms are accidentally ingested, the digestive fluid in the stomach will digest these mushrooms. After poisoning has occurred, only the vomit or feces of patients are usually obtained. These conditions may degrade the mushroom DNA to varying degrees. Therefore, to validate the applicability of the LAMP primer set in these situations, we simulated the cooking and human digestion processes of mushrooms by boiling and digesting mushrooms before LAMP testing. Results are shown in Tables 6 and 7 and Figures 6, 7. Except for the blank control, all samples were positive, showing that the LAMP primer set designed in this study can detect the target species (i.e., *G. infula*) from boiled and digested single and mixed mushroom species (*G. infula* content low up to 1%). The above applicability test results were all obtained on the basis of three repeated experiments.

**Table 6:** Applicability results of LAMP assay with boiled mushrooms.

Number	Boiled samples	LAMP results
1	*Gyromitra infula*	+
2	50% *Gyromitra infula* + 50% *Morchella esculenta*	+
3	10% *Gyromitra infula* + 90% *Morchella esculenta*	+
4	1% *Gyromitra infula* + 99% *Morchella esculenta*	+
0	ddH_2_O	−

“+” is positive and “−” is negative.

**Table 7:** Applicability results of LAMP assay with digested mushrooms after boiled.

Number	Digested samples after boiled	LAMP results
1’	*Gyromitra infula*	+
2’	50% *Gyromitra infula* + 50% *Morchella esculenta*	+
3’	10% *Gyromitra infula* + 90% *Morchella esculenta*	+
4’	1% *Gyromitra infula* + 99% *Morchella esculenta*	+
0	ddH_2_O	−

“+” is positive and “−” is negative.

## Discussion

*Gyromitra infula* is distributed in different regions of China in summer and autumn and is similar to some edible saddle mushrooms, such as *Helvella macropus*. Humans may confuse *G. infula* with them, thereby developing mushroom poisoning. The technology of LAMP can be used for rapid detection of *G. infula* and is a simple method. The species-specific amplification region is an essential challenge in designing efficient LAMP primer sets (Tomita et al., 2008). Schoch et al. (2012) amplified SSU, LSU, ITS, RPB1, RPB2, and MCM7 fragments during fungal molecular identification and found that ITS sequence was highly consistent in intraspecies, and significantly different in interspecies, and it was easy to conduct PCR amplification. Finally, they proposed using the ITS gene as the target gene fragment for fungal amplification. Subsequently, they obtained extensive support, and ITS was used for fungal DNA barcoding studies (Garnica et al., 2016; Badotti et al., 2017; Wang et al., 2019). Simultaneously, in consideration that sequences of many *Gyromitra* species are present in the NCBI GenBank database, we selected the ITS gene sequence as an amplification target to design *G. infula* LAMP-specific primer sets. In the selection of LAMP primer design sites, to ensure the specificity of the primers, there should be a certain number of mismatches between the sequence bases of the target species and the closely related species to reduce nonspecific amplification. For example, Xiong et al. (2020) found mismatch sites between *Oncorhynchus mykiss* and closely related species when designing LAMP primers for *O. mykiss* so that the primers were highly specific for *O. mykiss*. The *G. infula* LAMP primer set I designed in this study had some mismatches with other *Gyromitra* species at the 3' end of G.mol-F3/B3 and the 5' end of G.mol-F1c/B1c, showing that the *G. infula* LAMP primers designed in this study had high specificity. Whereas, the control primer set II presented non-specificity, and the main reason is there are not sufficient numbers of mismatches with closely related species, resulting in presence of binding sites with non-target species.

Recently, various LAMP visualizable detection methods have continuously emerged, and the most commonly used visualizable method is turbidimetry and colorimetry (Tomita et al., 2008; Denschlag et al., 2013). Turbidimetry is simple to operate and low-cost, but the sensitivity is low. Furthermore, it is difficult to see the results when the low reactant concentration (Njiru et al., 2008). Currently, colorimetry is widely accepted. Examples include methods with results that can be observed with the naked eye, such as the calcein fluorescence method, hydroxynaphthol blue/eriochrome black T staining method, pH indicator method. During the reaction, the reaction tubes do not need to be opened, which reduces aerosol contamination and false positive rate (Goto et al., 2009). In this study, the pH indicator method was used (Daskou et al., 2019, 2021; Shi et al., 2021), such that a chromogenic agent was added to the LAMP reaction system (such as Warm Start LAMP master mix), and the changes in the colorimetric result were used for interpretation to observe whether red-yellow color reaction occurred during product amplification. A yellow color means that the result is positive, that the target species DNA or the target species component is present in the tested sample. A red color means that the result is negative, that the target species DNA is absent. The pH indicator of phenol red was used as a dye in the reaction system. As LAMP amplification occurred, Bst conducted polymerization, and the number of protons in the reaction mix was changed, thereby changing the pH and causing the solution color to change from pink to yellow.

Currently, in addition to LAMP method established in this study, *G. infula* can be identified by other methods, such as traditional morphology identification, routine PCR, and so on. Extensive morphology features and professional mycologists are required to accurately identify species in traditional morphology method. Whereas, in mushroom poisoning incidents, the morphology of mushrooms will change greatly because of procession, such as cooking or digesting, which is hard to get correct identification results based on morphology. Moreover, compared to routine PCR, the LAMP method in this study has a higher specificity and sensitivity due to its specific primer set designed based on six specific binding sites of *G. infula*. Additionally, the LAMP method does not require complex heating and cooling processes, cumbersome electrophoresis, and ultraviolet observation, which have distinct advantages over routine PCR. Furthermore, although some other methods, such as real-time fluorescence PCR, PCR-restriction fragment length polymorphism (PCR-RFLP), and the liquid chromatography-mass spectrometry (LC–MS, used for the detection of toxins) might be used for identification of *G. infula*, they rely on expensive instruments, a long detection cycle, high detection costs, and high sample pretreatment, which are not suitable for application in resource-poor areas and on-site detection. Therefore, the present LAMP method with abovementioned advantages is superior to other methods and is suitable for identifying *G. infula*. However, we need to pay attention to controlling the content of the DNA template in the reaction system because the larger amount of DNA template can have an inhibitory effect on the LAMP reaction (Njiru et al., 2008; Wang et al., 2021) presumably due to the negative influence of polyphenols and polysaccharides contained in mushrooms (Newbury and Possingham, 1977; Fang et al., 1992).

## Conclusion

In this study, we established a visual and rapid LAMP method for the identification of *G. infula*. The results showed that this method can accurately and specifically identify poisonous *G. infula* within 90 min. The test results can be directly observed with the naked eyes, which can be used for rapid onsite detection. Meanwhile, high sensitivity for the LAMP method with the detection limit of 1 ng was confirmed in this study. The method can be conducted without expensive and sophisticated instruments, which is suitable for resource-poor areas. Furthermore, the boiled and/or digested *G. infula* were successfully detected in this study, indicating its strong adaptability. Therefore, the LAMP method established in this study is accurate, visual, rapid, sensitive, adaptable, and low-cost for the identification of *G. infula*. Using our method, an on-site detection kit of *G. infula* can be developed for clinical treatment and forensic analysis. In the future, increasing methods that are more rapid, more efficient, simpler and more low-cost will be explored for identifying varied poisonous mushrooms, further used for diagnosis, targeted treatment and prevention of poisoning.

## Data Availability Statement

The datasets presented in this study can be found in online repositories. The names of the repository/repositories and accession number(s) can be found in the article/supplementary material.

## Author Contributions

ET designed the experiments and morphologically identified the species collected in this study. YF provided some specimens and photos of *Gyromitra*, *Morchella*, *Helvella*, and *Verpa* species. XX, RD, BL, CG, and YZ completed the analysis ITS sequences of all specimens and LAMP assay. XX and ET wrote the manuscript. XX, BL, YF, RD, CG, YZ, and ET contributed to collection of specimens used in this study. All authors contributed to the article and approved the submitted version.

## Funding

This research was supported by the National Key Research and Development Program of China (2019YFC1604703) and National Natural Science Foundation of China (no. 31860009).

## Conflict of Interest

The authors declare that the research was conducted in the absence of any commercial or financial relationships that could be construed as a potential conflict of interest.

## Publisher’s Note

All claims expressed in this article are solely those of the authors and do not necessarily represent those of their affiliated organizations, or those of the publisher, the editors and the reviewers. Any product that may be evaluated in this article, or claim that may be made by its manufacturer, is not guaranteed or endorsed by the publisher.

## Footnotes

^1^ https://blast.ncbi.nlm.nih.gov/Blast.cgi

^2^ https://cgm.sjtu.edu.cn/GLAPD/online/

^3^ https://www.ncbi.nlm.nih.gov/tools/primer-blast/
